# Circ-RNA Expression Pattern and circ-RNA-miRNA-mRNA Network in The Pathogenesis of Human Intervertebral Disc Degeneration

**DOI:** 10.22074/cellj.2021.6832

**Published:** 2021-05-26

**Authors:** Zhiliang Guo, Yuanyuan Liu, Yu Gao, Xiumei Guan, Hong Li, Min Cheng

**Affiliations:** 1 Department of Orthopedic, No. 89 Hospital of Chinese PLA, Weifang, 261021, China; 2 Stomatology Medical College, Weifang Medical University, Weifang 261053, Shandong, China; 3 Clinical Medical College, Weifang Medical University, Weifang 261053, Shandong, China

**Keywords:** Biomarkers, Circular, Intervertebral Disc Degeneration, RNA

## Abstract

**Objective:**

The present study aimed to screen the differentially expressed (DE) circular RNAs (circ-RNAs) between lumbar intervertebral disc degeneration (IVDD) and normal tissues.

**Material and Methods:**

In this experimental study, microarray hybridization was performed to evaluate circ-RNA expression, and the DE circ-RNAs were confirmed by quantitative real-time polymerase chain reaction (qRT-PCR). Host genes of DE circ-RNAs were predicted, and their functions were evaluated. Further, a competitive endogenesis (ce) RNA network among 4 DE circ-RNAs-miRNA-mRNA was constructed by Cytoscape.

**Results:**

A total of 2636 circ-RNAs were detected in all samples; among them, 89.23% were exonic circ-RNAs. There were 138 DE circ-RNAs, including 134 up-regulated circ-RNAs and 4 downregulated circ-RNAs in IVDD samples. qRT-PCR validation experiments showed that expression trends of hsa_circ_0003239, hsa_circ_0003162, hsa_circ_0005918, and hsa_circ_0005556 were in line with the microarray analysis results. Functional enrichment analysis showed that host genes of DE circ-RNAs significantly disturbed pathways of regulation of actin cytoskeleton, propanoate metabolism, and ErbB signaling pathway. The four DE circ-RNAs related ceRNA network was constructed.

**Conclusions:**

Our results revealed that circ-RNAs can function as miRNA sponges and regulate parent gene expression to affect IVDD.

## Introduction

Human lumbar intervertebral disc degeneration (IVDD) disease contributes a lot to low back pain (1). Numerous studies have indicated that a variety of cellular events are disrupted in the progression of IVDD, ranging from matrix synthesis to cytokine expression (2). Although increasing evidence has revealed that IVDD is a multifaceted spinal disease, many studies have confirmed that the primary factors contributing to IVDD are genetic factors (3, 4).

Circular RNAs (circ-RNAs) are newly defined non-coding RNAs with special structures (5-7). Unlike linear RNA, which terminates with the 5’ cap and 3’ tail, circRNA forms covalently closed continuous loop structures and are considered as evolutionarily highly conservative and stable (8-10). Increasing evidence suggests that circ-RNAs are present in nearly all types of species and expressed in a tissue- and disease-dependent manner (11, 12). Therefore, circ-RNAs might more appropriate to be used as a molecular diagnostic biomarker for various diseases, including colon cancer, ovarian cancer, and gastric cancer (13-18). 

Studies on circ-RNAs are in their early stages. Several studies have shown that circ-RNAs are involved in IVDD diseases and have determined their expression profiles (19, 20). Wang et al. (21) demonstrated that circ-RNAs regulated the viability, degradation, apoptosis, and oxidative stress in nucleus pulposus (NP) cells. However, the role of circ-RNAs in lumbar discs and their overall contribution to IVDD pathogenesis are few investigated. Recent studies found that circ-RNAs can efficiently bind to miRNA and regulate downstream mRNA expression indirectly; these were termed as “competitive endogenesis (ce) RNA” (22). In a recent study, Cheng et al. demonstrated that circRNA VMA21 protects against IVDD through targeting miR-200c and X linked inhibitor-of-apoptosis protein (23). Circ-RNA_104670 functions as a ceRNA by binding miR-17-3p to regulate the expression of MMP2 during NP degradation (24). Circ-4099 functions as a ceRNA by blocking miR-616-5p inhibition of Sox9 in IVDD (25). These studies suggested circ-RNAs can act as ceRNAs to regulate the pathological process of IVDD. Therefore, in this study, we performed acirc-RNA microarray to screen the DE circ-RNAs that might regulate the viability and functions of NP cells. Quantitative reverse transcription-polymerase chain reaction (qRT-PCR) was performed to validate the microarray results. Besides, a ceRNA network of circ-RNA-miRNA-mRNA was constructed. Our study could provide novel data for IVDD diagnosis and pathogenesis.

## Materials and Methods

### Human nucleus pulposus sample collection

In this experimental study, NP tissues from degenerative lumbar and normal lumbar were collected. The patient demographics and IVDD grading were also collected. Lumbar disc tissue (three lumbar disc tissues and three normal tissues) was isolated from surgical operations, immediately put into liquid nitrogen. This study was approved by the Human Ethics Committees Review Board at No. 89 Hospital of Chinese PLA (No.1893), Weifang, China. The written informed consent was obtained from all participants.

### Microarray and quantitative analysis 

Total RNA of samples was extracted by TRIzol reagent (Invitrogen, Carlsbad, CA, USA) and quantified using a NanoDrop ND-1000 (NanoDrop, Wilmington, DE, USA). Sample preparation and microarray hybridization were performed using Array star standard protocols, as indicated in previous studies (26, 27). Raw microarray data extraction and analysis were performed using the R software package (version 2.15, http://www.rproject.org/). First, the data were normalized and log2- transformed. Then, the DE circ-RNAs between IVDD and normal samples were identified by a t test based on the thresholds of fold-change ≥2.0 and P<0.05. Further, heat map, volcano plot, and MA plot were drawn to display circ-RNA expression patterns among samples.

### Validation of differentially expressed circ-RNAs using quantitative real-time polymerase chain reaction

The DE circ-RNAs in the microarray experiments were further confirmed by qRT-PCR using the same samples of circ-RNA microarray. Five DE circ-RNAs were selected to verified based on their significant differences and raw signal intensity of expression. β-actin as used as an internal control. Total RNA was isolated and was reverse-transcribed to cDNA using the SuperScript III First-Strand synthesis system (Life Technologies, Carlsbad, CA, USA). Further, the expression of the 5 DE circ-RNAs was determined on the ABI7500 instrument (Thermo Fisher Scientific, Waltham, MA, USA) using the SYBR Green I kit (Thermo Fisher Scientific, Waltham, MA, USA) with primers listed in Table 1. All qRT-PCRs were conducted in triplicate.

**Table 1:** The primer sequence used in quantitative real-time polymerase chain reaction


Gene	Primer sequences (5´-3´)	Annealing temperature (˚C)	Product sizes (bp)

*β-actin (HUMAN)*	F: AGCACAGAGCCTCGCCTTTG	60	208
	R: CTTCTGACCCATGCCCACCA		
*circ_0003239*	F: CCAAGAGACTGCTTTTGAGTGACA	60	124
	R: TTTTAGGAGGTCGGAGGGGATA		
*circ_0005556*	F: GATGGACTGGTTCGCTTGGT	60	149
	R: TTTCGTGATGATAAAGGATGCA		
*circ_0003162*	F: CTCAGGAACCTTGGGTAATGTG	60	231
	R: CCACTATTGTCAACATTAGCCAGA		
*circ_0075504*	F: ATCTTTGGACTGACTGTGGCACT	60	202
	R: GCATCCAGTTATTAGGTAGCCAAA		
*circ_0005918*	F: GCAAGGAATGATTATCTTCTTACCC	60	187
	R: GAGCCATCTGTTCAGTCTCAAAGT		


### GO and KEGG pathway analysis for differentially expressed circ-RNAs related to intervertebral disc degeneration

Co-expression between DE circ-RNAs and mRNAs was calculated, and a gene co-expression network was built using Cytoscape (version 3.0). The co-expressed mRNAs of DE circ-RNAs were regarded as their host genes. The functions of DE circ-RNAs were predicted by gene oncology (GO) enrichment analysis on their host genes in terms of biological processes (BP), cellular components (CC), and molecular functions (MF). Biological pathways involved by the DE circ-RNAs were predicted by the Kyoto Encyclopedia of Genes and Genomes (KEGG) (http://www.genome. jp/kegg/) analysis. Both GO and KEGG enrichment analyses were performed using Database for Annotation, Visualization, and Integrated Discovery (DAVID; http://www.david.abcc.ncifcrf.gov/) (28) based on criteria of P<0.05.

### Construction of circ-RNAmiRNA-mRNA network

The potential miRNAs binding with DE circ-RNAs were predicted by Array star’s in-house miRNA target prediction software based on TargetScan and miRanda (29, 30). A circ-RNA-miRNA-mRNA network was then visualized using Cytoscape v3.0. Five confirmed circ-RNAs, hsa_circ_0003239, hsa_circ_0003162, hsa_ circ_0005918, hsa_circ_0075504, and hsa_circ_0005556, were annotated in detail based on the circ-RNA-miRNA-mRNA interaction network. 

### Statistical analysis

The statistical analysis of microarray data was performed by the R software package (version 2.15, http://www.r-project.org/). The statistical analysis of qRT-PCR was performed using SPSS (version 13.0) software (SPSS, Inc., Chicago, IL, USA). Differences between the two groups were analyzed using the t test, and data were reported as the mean ± standard deviation (SD). P values of less than 0.05 were considered significant. 

## Results

### Screening of differentially expressed circ-RNA in intervertebral disc degeneration

**Fig.1:**
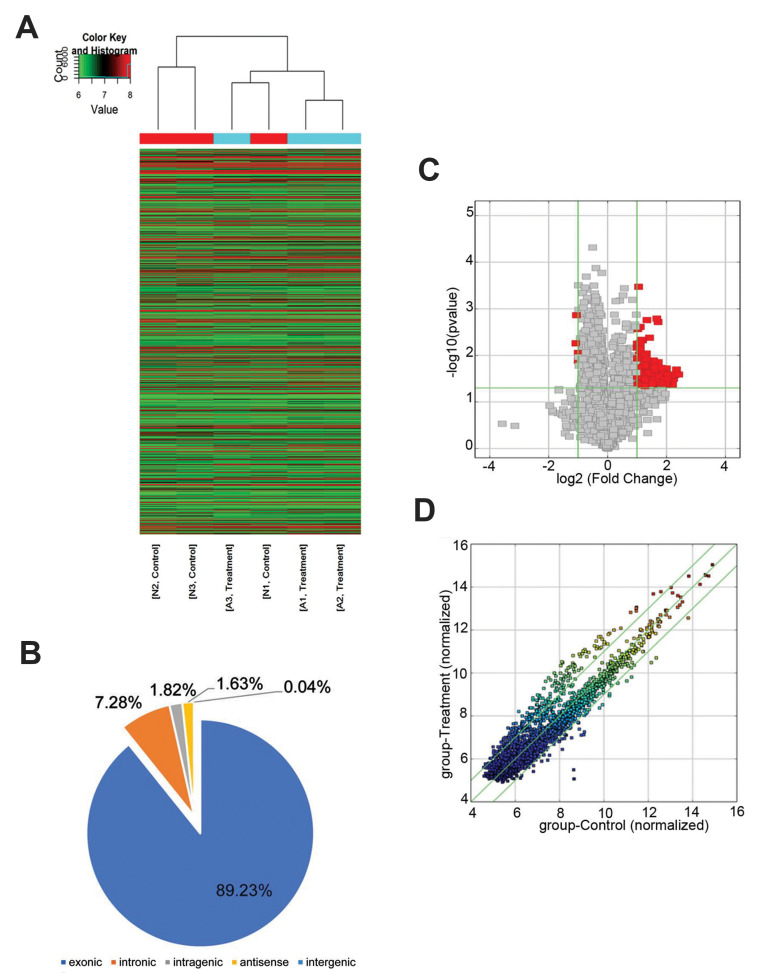
The circ-RNA microarray results. **A.** Exonic circ-RNAs accounted for 89.23%, followed by 7.28% intronic circ-RNAs, and 1.82% intragenic circ-RNAs.** B.** Heat map showed the circ-RNAs detected in all samples. The row of colored boxes indicated circ-RNAs, and the column indicated sample names. **C.** Volcano plot and **D.** Scatter plot showing the DE circ-RNAs. DE; Differentially expressed and circ-RNAs; Circular RNAs.

A total of 2636 circ-RNAs were detected by Arraystar Human circ-RNA Array (Fig .1A). The results suggested that the circ-RNAs consisted of 89.23% exonic circ-RNAs (2352 circ-RNAs), 7.28% intronic circ-RNAs (192 circ-RNAs), 1.82% intragenic circ-RNAs (48 circ-RNAs), 1.63% antisense circ-RNAs (43 circ-RNAs) and 0.04% intergenic circ-RNAs (1 circ-RNA) (Fig .1B). There were 134 up- and four down-regulated circ-RNAs in degenerative lumbar NP samples compared with normal control samples with the criteria of fold change ≥2.0 and P<0.05 (Fig .1C, D).

### Validation results of selected circ-RNAs by quantitative real-time polymerase chain reaction

**Fig.2:**
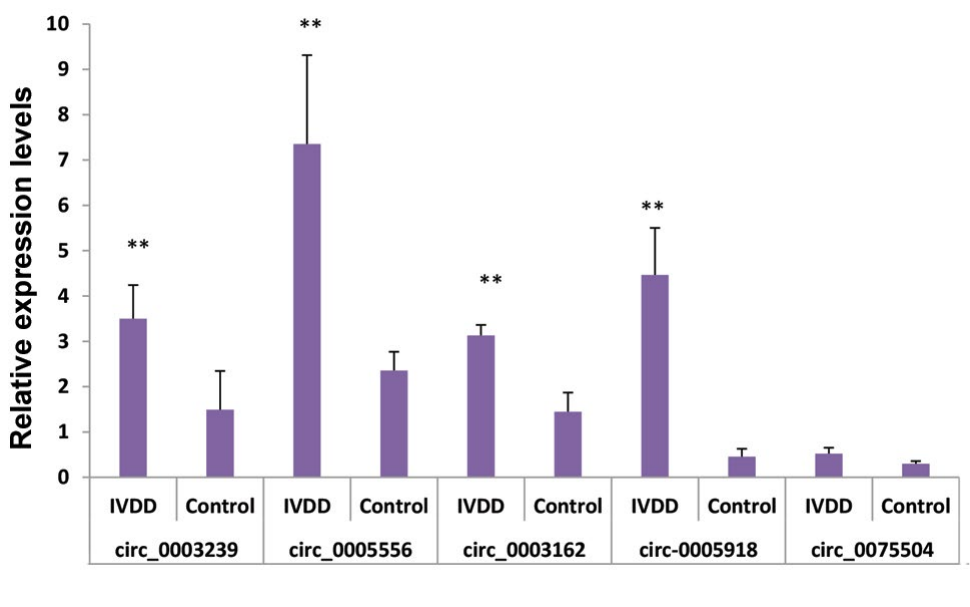
The expression levels of 5 DE circ-RNAs were validated by qRT-PCR. Each qRT-PCR assay was performed in triplicate. **; P<0.01, DE; Differentially expressed, circ-RNAs; Circular RNAs, qRT-PCR; Quantitative real-time polymerase chain reaction, and IVDD; Intervertebral disc degeneration.

Since the microarray data might generate some false-positive results, we further verified the microarray results by qRT-PCR using the same samples. Exonic circ-RNAs were chosen based on their significant differences and raw signal intensities of expression. Five circ-RNAs (hsa_ circ_0003239, hsa_circ_0005556; hsa_circ_0003162; hsa_circ_0075504; and hsa_circ_0005918) that were up-regulated in the IVDD lumbar nucleus by 3.52, 5.05, 5.33, 4.87, and 4.69-fold, respectively in the microarray results were selected for validation. As shown in Figure 2, the relative expression levels of four circ-RNAs (hsa_ circ_0003239, hsa_circ_0003162, hsa_circ_0005918, and hsa_circ_0005556) in qRT-PCR results were in line with those in the microarray results. The objective circ-RNA validation rate was 80%, suggesting that the microarray results were reliable.

### Functional annotation of differentially expressed circ-RNAs related to intervertebral disc degenera

**Fig.3:**
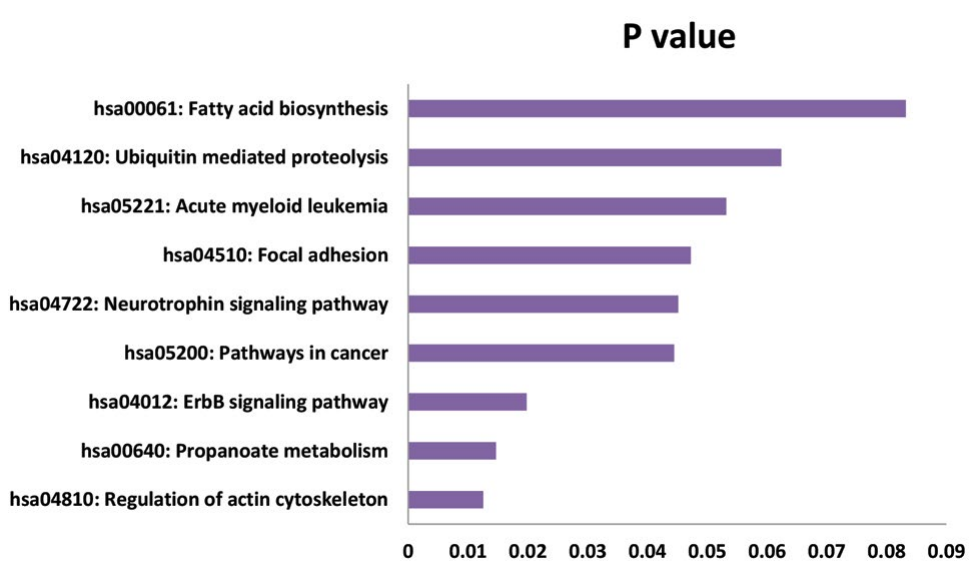
Gene ontology analysis of the host genes that shows the up-regulated circ-RNAs based on the DAVID database.

**Fig.4:**
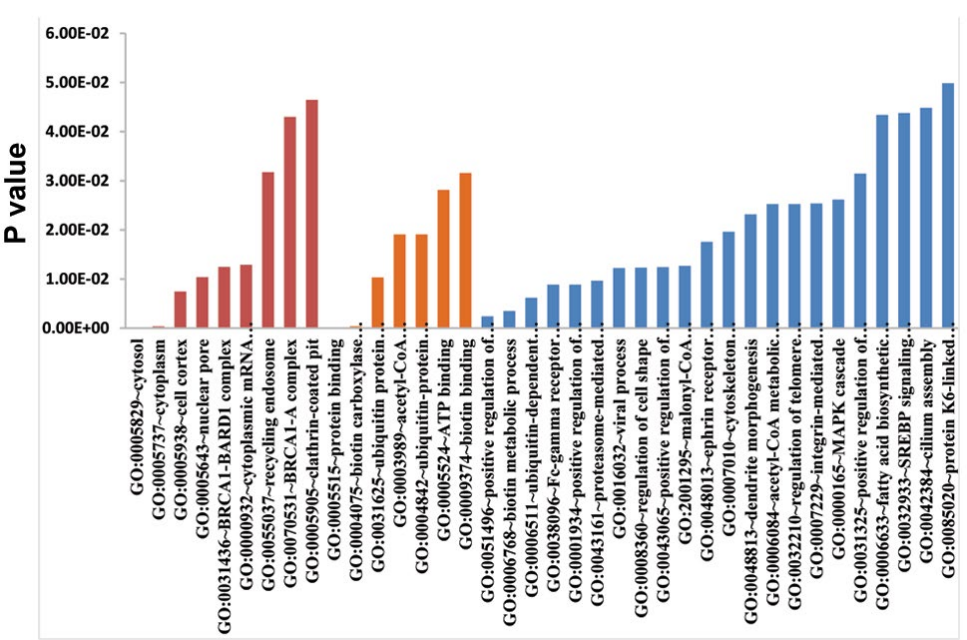
KEGG enrichment analysis of the host genes that demonstrates the up-regulated circ-RNAs according to the DAVID database.

The host genes of DE circ-RNAs were predicted by gene co-expression analysis, and GO and KEGG pathway analyses were performed to investigate the functional annotation of host genes of DE circ-RNAs related to IVDD. At the criteria of P<0.05, 8 GO-CC terms, 6 GO-MF terms, and 22 GO-BP terms were significantly enriched by up-regulated circ-RNAs (Fig .3). The results suggested the circ-RNAs were located in cytosol, cytoplasm, and cell cortex and significantly involved in BP of positive regulation of stress fiber assembly (host genes of circBRAF, circPAK1, circLPAR1 and circARHGEF10L, P=0.02411), biotin metabolic process (host gens of circACACA, circACACB, and circPCCA, P=0.003482), ubiquitin-dependent protein catabolic process (host genes of circCUL3, circNPLOC4, circCUL4A, circUBE2G1, circUSP34, and circFBXO7, P=0.006181) and Fc-gamma receptor signaling pathway involved in phagocytosis (host genes of circACTR2, circMYO10, circPTK2, circDOCK1 and circPAK1, P=0.008847). KEGG pathway enrichment analysis found that the host genes of DE circ-RNAs significantly disturbed pathways of regulation of actin cytoskeleton (host genes of circARHGEF4, circPTK2, circDOCK1, circBRAF, circITGA3 and circPAK1, P=0.01254), propanoate metabolism (host genes of circACACA, circACACB, and circPCCA, P=0.01468) and ErbB signaling pathway [host genes of circPTK2, circBRAF, circSTAT5B, circPAK1, P=0.01982 (Fig .4)].

### Construction of circ-RNA-miRNA-mRNA network

**Fig.5:**
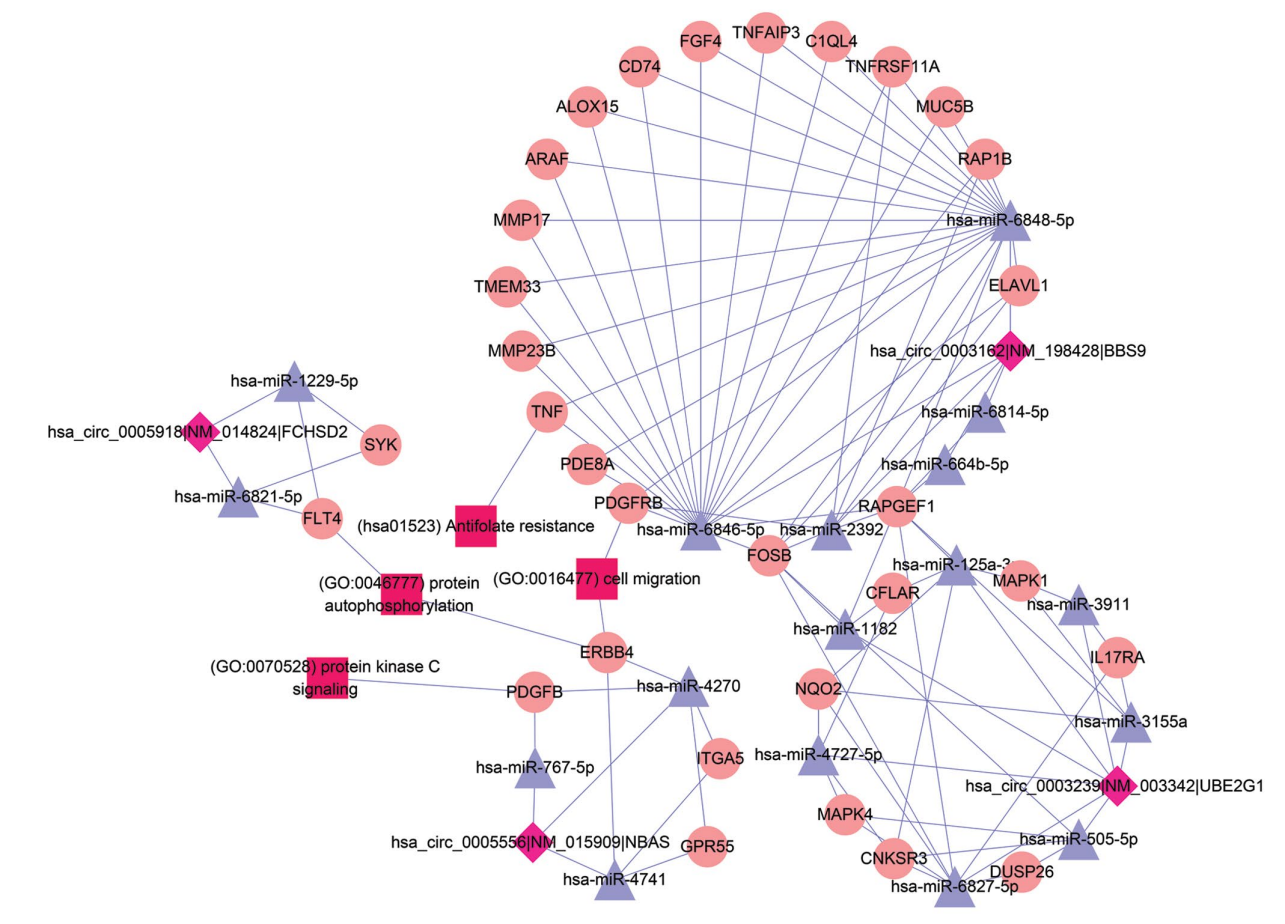
DAVID construction of the circ-RNA-miRNA mRNA network. Diamond nodes represent circ-RNAs, and purple triangle nodes represent miRNAs.

The miRNAs and mRNAs related to the four qRT-PCR-verified DE circ-RNAs, including hsa_circ_0003239, hsa_circ_0003162, hsa_circ_0005918, and hsa_ circ_0005556, were constructed. The four DE circ-RNAs regulated 31 mRNAs by competitive binding with 17 miRNAs (Fig .5). Notably, hsa_circ_0003162 regulated 18 mRNAs by competitive binding with hsa-miR-6848- 5p, hsa-miR-6846-5p, hsa-miR-2392, hsa-miR-664B-5p and hsa-miR-6814-5p.

## Discussion

Studies have shown that many abnormal cell events occur in the process of IVDD, such as the increase in NP cell apoptosis, various inflammatory factors, and matrix metalloproteinase expression (31, 32). This series of changes suggest that specific molecular gene expression in the intervertebral disc is dysfunctional, and the corresponding regulatory factors are altered, but the etiology and the precise mechanism of disc degeneration remain unclear. In this study, we performed acirc-RNA microarray on human normal and degenerative lumbar NP samples and identified 138 DE circ-RNAs in intervertebral discs from IVDD and normal tissues. Five DE circ-RNAs were selected, and four circ-RNAs (hsa_ circ_0003239, hsa_circ_0003162, hsa_circ_0005918, and hsa_circ_0005556) were successfully validated by qRT-PCR, showing consistent results with microarray. The four DEcirc-RNAs regulated 31 mRNAs by competitive binding with 17 miRNAs in the ceRNA network. Notably, hsa_circ_0003162 regulated 18 mRNAs by competitive binding with hsa-miR-6848-5p, hsa-miR-6846-5p, hsa-miR-2392, hsa-miR-664B-5p and hsa-miR-6814-5p. A study of gastric cancer showed that hsa_circ_0005556 and hsa_circ_0003239 attenuate gastric cancer proliferation and metastasis (33). However, the other DE circ-RNAs were not investigated previously. Although the specific functions of most circ-RNAs remain unclear, accumulating evidence has revealed the role of circ-RNAs as miRNA sponges (34, 35). We speculated these ceRNA relationships were important in the occurrence and progression of IVDD. However, further experiments are warranted to validate these relationships.

Functional enrichment analyses suggested that the host genes of the upregulated circ-RNAs were significantly involved in BP of positive regulation of stress fiber assembly, biotin metabolic process, ubiquitin-dependent protein catabolic process and Fc-gamma receptor signaling pathway involved in phagocytosis as well as pathways of regulation of actin cytoskeleton, propanoate metabolism, and ErbB signaling pathway. Stress fibers are contractile actomyosin bundles found in many cultured non-muscle cells, where they have a central role in cell adhesion and morphogenesis (36). The pathologic findings in IVDD include protrusion, spondylolysis, and/or subluxation of vertebrae (spondylolisthesis) and spinal stenosis. We hypothesized that stress fiber assembly might play a role in protrusion. Besides, a previous study suggested the ErbB signaling pathway was disturbed in IVDD, which is consistent with our study.

The strengths of this study are that the circ-RNA microarray of IVDD samples generated hundreds of DE circ-RNAs that might play essential roles in IVDD development. However, there are some limitations to this study. First, the sample size of circ-RNA is relatively small; only three samples in IVDD groups and three samples in the control group were evaluated. Second, though we constructed a ceRNA network for the verified DE circ-RNAs, these ceRNA relationships were not verified by further in vitro or in vivo experiments. In our further studies, we will perform experiments to validate the ceRNA relationship in the DE circ-RNAs-miRNA-mRNA network.

## Conclusion

The circ-RNA microarray detected 2636 circ-RNAs expression, with 134 upregulated circ-RNAs and four down-regulated circ-RNAs in IVDD samples. The qRT-PCR validation experiments showed that hsa_ circ_0003239, hsa_circ_0003162, hsa_circ_0005918, and hsa_circ_0005556 expression levels were consistent with the microarray analysis results. Our results revealed more circ-RNAs that play important roles in IVDD development.
